# Small molecule-induced epigenomic reprogramming of APL blasts leading to antiviral-like response and c-MYC downregulation

**DOI:** 10.1038/s41417-022-00576-w

**Published:** 2022-12-19

**Authors:** Stefano Amatori, Giuseppe Persico, Francesco Cantatore, Martina Rusin, Mauro Formica, Luca Giorgi, Eleonora Macedi, Francesca Casciaro, Alfredo Errico Provenzano, Stefano Gambardella, Roberta Noberini, Tiziana Bonaldi, Vieri Fusi, Marco Giorgio, Mirco Fanelli

**Affiliations:** 1 Molecular Pathology Laboratory “PaoLa”, Department of Biomolecular Sciences, University of Urbino Carlo Bo, Fano, PU Italy; 2 Department of Experimental Oncology, IRCCS, European Institute of Oncology, Milan, Italy; 3 Department of Pure and Applied Sciences, University of Urbino Carlo Bo, Urbino, Italy; 4 Department of Biomedical Sciences, University of Padua, Padua, Italy; 5 Department of Oncology and Hematology-Oncology, University of Milan, Milan, Italy

**Keywords:** Gene expression analysis, Leukaemia

## Abstract

Acute promyelocytic leukemia (APL) is an aggressive subtype of acute myeloid leukemia (AML) in which the PML/RARα fusion protein exerts oncogenic activities by recruiting repressive complexes to the promoter of specific target genes. Other epigenetic perturbations, as alterations of histone H3 lysine 9 trimethylation (H3K9me3), have been frequently found in AMLs and are associated with leukemogenesis and leukemia progression. Here, we characterized the epigenomic effects of maltonis, a novel maltol-derived molecule, in APL cells. We demonstrate that maltonis treatments induce a profound remodulation of the histone code, reducing global H3K9me3 signal and modulating other histone post-translational modifications. Transcriptomic and epigenomic analyses revealed that maltonis exposure induces changes of genes expression associated with a genomic redistribution of histone H3 lysine 4 trimethylation (H3K4me3) and lysine 27 acetylation (H3K27ac). Upregulation of interferon alpha and gamma response and downregulation of c-MYC target genes, in function of c-MYC reduced expression (monitored in all the hematopoietic neoplasms tested), represent the most significant modulated pathways. These data demonstrate the ability of maltonis to epigenetically reprogram the gene expression profile of APL cells, inducing an intriguing antiviral-like response, concomitantly with the downregulation of c-MYC-related pathways, thus making it an attractive candidate for antileukemic therapy.

## Introduction

Epigenetic modifications (e.g., DNA methylation and histone post translational modifications—histone PTMs) are crucial for the proper regulation of gene expression and the establishment of genomic stability, and their dysregulation is a common feature in human cancers and is thought to play an important role also in leukemia pathogenesis [1]. In fact, inappropriate gene silencing of precursor cells in the hematopoietic compartment may be part of the mechanisms of hematopoietic dysfunction, and of leukemogenesis as well [2, 3].

Acute myeloid leukemia (AML) represents the most common, and one of the most aggressive, form of hematopoietic disorders in adults and is characterized by a genetic heterogeneity that, in several cases, includes chromosomal translocations that lead to the expression of chimeric proteins with key biological properties. Acute promyelocytic leukemia (APL), one of the best studied forms of AML, is characterized in ~95% of cases by the chromosomal translocation t(15:17), responsible for the generation of the PML/RARα oncoprotein and the consequent block of blasts differentiation processes [4, 5]. In addition to the mechanisms by which PML/RARα exerts its biological activities, through the recruitment of repressive complexes to the target gene promoters, epigenetic perturbations have been described in AMLs and are considered to play an important role in leukemogenesis and leukemia progression [6]. Changes in the genomic distribution of both DNA methylation and histone PTMs are associated with AML and different therapeutic strategies have been developed to target the anomalous epigenetic status of the blasts [7, 8]. Among histone PTMs, the histone 3 lysine 9 trimethylation (H3K9me3), known to be involved in the formation of transcriptionally silent heterochromatin, is found to be highly regulated in normal human stem cells (HSC) differentiation and self-renewal alongside the establishment of pre-leukemic stem cells (LSC) [9, 10]. In general, it has been demonstrated that AMLs are characterized by perturbations of H3K9me3 distribution that may be also related to the development of drug-resistance [9, 11, 12]. In accordance with this observation, H3K9me3 modifying enzymes, such as SETDB1 and KDM3B, showed key roles in the survival of AML cells and in APL progression [6, 13].

Furthermore, in AML patients, alterations of H3K9 methylation were found associated with hundreds of promoter regions and related with inactivation of tumor suppressor genes, as well as with the outcome of the disease in >70% of cases [9, 14, 15].

Conversely, loss of H3K9me3 methyltransferases has a protective role in some models of leukemia, probably due to the de-silencing of tumor suppressor genes, and the inhibition the H3K9me3 methyltransferase SUV39H1 has been proposed as a potential therapeutic strategy [16, 17]. Given the contribution of H3K9 trimethylation in leukemogenesis, and considering its reversible nature, this histone PTM represents indeed an attractive therapeutic target in AML.

**Fig. 1:**
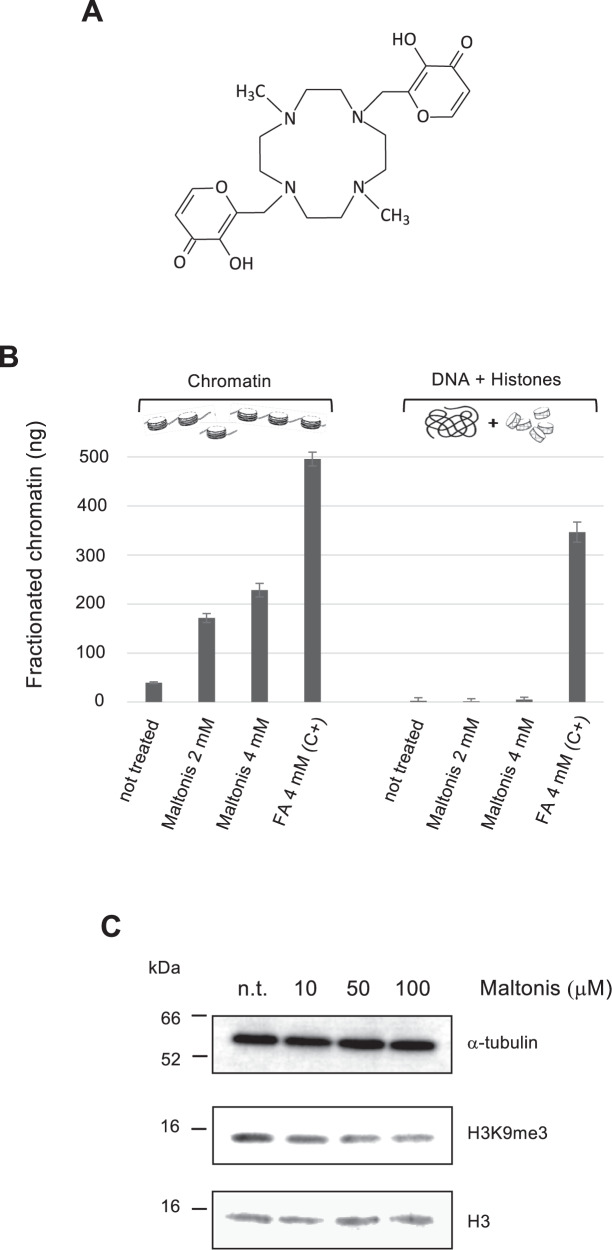
Maltonis-mediated cell-free chromatin complexes formation and H3K9me3 reorganization. **A** Maltonis structure. **B** DNA-histones cross-linking induced by maltonis. Native chromatin or free DNA plus histone proteins were incubated for 4 h at 37 °C with maltonis or with formaldehyde (FA) at the concentrations reported in figure. DNA-protein complexes were then isolated by biochemical fractionation and the DNA in chromatin-containing fractions was fluorimetrically quantified. C + (FA 4 mM): positive control. The experiment was conducted in triplicate and the statistical significance of each experimental condition, using chromatin or DNA and histones, was evaluated using student’s t-test (two-sided), *p* value < 0.01. **C** Evaluation of global H3K9me3 changes induced by maltonis in NB4 cells. NB4 cells were treated with the reported concentrations of maltonis and total cell lysates were analyzed by western blot.

The biological activity of maltonis, a synthetic maltol-derived molecule belonging to the class of hydroxypyrones (Fig. 1A), has been described both in vitro [18] and in vivo [19]. Preliminary mechanistically observations revealed the ability of maltonis to interfere with the chromatin structure [20] inducing a DNA damage-like response characterized by γ-H2AX recruitment [19].

Here, the effects of maltonis on the histone code and the associated remodulation of the gene expression were investigated in the NB4 cell line, considered the most representative in vitro cellular model of human APL. We report the ability of maltonis to induce massive changes in the distribution of H3K9me3, histone 3 lysine 4 trimethylation (H3K4me3), and histone 3 lysine 27 acetylation (H3K27ac), concomitantly with a consistent reorganization of gene expression programs. The biological response to maltonis was finally characterized, observing a peculiar antiviral response concomitantly with a massive c-MYC pathway downregulation.

## Materials and methods

### Biochemical fractionation

Nuclei preparation and native chromatin isolation were carried out as already described [21]. Genomic DNA was purified from an aliquot of isolated native chromatin with the PCR Purification Kit (QIAGEN, Hilden, Germany) following manufacturer instructions, while histone proteins were extracted following the procedure previously described by Shechter and colleagues [22].

Native chromatin, free genomic DNA plus histone proteins, or free genomic DNA alone, were incubated for 4 h at 37 °C with maltonis or with formaldehyde (as positive control of cross-linking) at the final concentrations of 2 mM and 4 mM, or left untreated. Afterward, biochemical fractionation was conducted in the presence of 2.5% SDS and 175 mM KCl as already described [20] and both precipitated (cross-linked fraction) and not precipitated (not cross-linked) chromatin fractions were fluorimetrically quantified by Qubit (ThermoFisher, Carlsbad, CA). The amount of precipitated DNA obtained using only free DNA, in all experimental conditions (not exposed or exposed to maltonis or formaldehyde), was considered as background and subtracted to the amount of DNA precipitated using native chromatin or a mix of free DNA and histone proteins.

### Cell culture, treatments, and western blotting

NB4, HL60, K562, Jurkat, and U937 cell lines were maintained in RPMI 1640 medium supplemented with 10% of fetal bovine serum (FBS, Gibco, Paisley, UK), 1% of L-glutamine (Lonza, Verviers, Belgium) and 1% of penicillin/streptomycin (Euroclone, Pero, MI, Italy) in a humidified atmosphere at 37 °C and 5% of CO_2_. Cell lines were originally obtained from ATCC repository and routinely tested by PCR method and MycoAlert (Lonza, Verviers, Belgium #LT07-318) for mycoplasma contamination by the European Institute of Oncology (Milan, Italy).

Maltonis was synthesized as already described [20] and used for the treatments of cells (stock solution of 10 mM diluted in distilled water) at the reported concentrations for 24 h [19]. Western blot analyses were performed as previously reported [23] using the following antibodies: anti-H3K9me3 (#39766, Active Motif, Carlsbad, CA, USA, 1:1000 dilution), anti-α-tubulin (#T9026, Sigma-Aldrich, Merck KGaA, Darmstadt, Germany, 1:500 dilution), anti-Histone H3 (#05-499, Sigma-Aldrich, Merck KGaA, Darmstadt, Germany, 1:500 dilution), anti-c-MYC (Y69, Abcam, Cambridge, MA, USA, 1:1000 dilution), and images acquired using Vü-C Imaging system (PopBio, Cambridge, UK).

### Histone PTMs mass spectrometry analysis

Histones were enriched from 2 × 10^6^ NB4 cells as previously described [24], in triplicate for each experimental condition. Approximately 4 µg of histone octamer were mixed with an equal amount of heavy-isotope labeled histones, which were used as an internal standard (super-SILAC mix) [25], and separated on a 17% SDS-PAGE gel. Histone bands were excised, chemically acylated with propionic anhydride and in-gel digested with trypsin, followed by peptide N-terminal derivatization with phenyl isocyanate (PIC) [26]. Peptide mixtures were separated by reversed-phase chromatography on an EASY-Spray column (Thermo Fisher Scientific), 25-cm long (inner diameter 75 µm, PepMap C18, 2 µm particles), which was connected online to a Q Exactive Plus instrument (Thermo Fisher Scientific) through an EASY-Spray™ Ion Source (Thermo Fisher Scientific), as described [26]. The acquired RAW data were analyzed using EpiProfile 2.0 [27], selecting the SILAC option, or manually [26]. For each histone-modified peptide, the % relative abundance (%RA) for the sample (light channel—L) or the internal standard (heavy channel—H) was estimated by dividing the area under the curve of each modified peptide for the sum of the areas corresponding to all the observed forms of that peptide and multiplying by 100. Light/Heavy (L/H) ratios of %RA were then calculated and are reported in Supplementary Table S1. The mass spectrometry data have been deposited to the ProteomeXchange Consortium [28] via the PRIDE partner repository with the dataset identifier PXD031448.

### RNA sequencing

RNA was isolated starting from 2 × 10^6^ cells using the RNeasy Mini Kit (QIAGEN, Hilden, Germany) and following manufacturer’s instructions, while RNA-seq libraries were prepared and sequenced in triplicate for each experimental condition, as already described [29].

Reads pre-processing and alignment was also performed as previously reported [29]. After removal of low expressed genes (25% lowest RPKM value), the remaining expressed genes (75% higher RPKM value, *n* = 17,989) were used to select the most 10% variable genes by which the relationships between samples have been investigated through a hierarchical clustering approach (Euclidian distance) using heatmap2 R package, or to perform principal component analysis (PCA). In addition, gene set enrichment analysis (GSEA) [30] using expressed genes (75% higher RPKM value, *n* = 17,989) was carried out by GSEA software, version 4.1.0, obtained from the Broad Institute (http://www.broadinstitute.org/gsea/downloads.jsp). HTseq counts were also used to measure the differential gene expression with the edgeR package, applying the TMM (Trimmed Mean of M-values) normalization. Genes were identified as differentially expressed (DEGs) when the following criteria were met: log2(fold change—FC) > 1.32, false discovery rate (FDR) < 0.01 and RPKM_Treat > 10 for upregulated DEGs, while log2(FC) < −1.32, FDR < 0.01, and RPKM_Crtl > 10 for downregulated DEGs. Where not specified, all plots are generated using ggplot2 R package.

### Chromatin immunoprecipitation sequencing (ChIP-seq)

ChIP procedure was conducted in triplicate for each histone PTM investigated, maltonis-treated and untreated NB4 cells were cross-linked for 10 minutes at 37 °C by adding, directly to the cell culture medium, 1/10 of cross-linking solution (50 mM Hepes—pH 7.5, 11% formaldehyde, 1 mM Na_2_EDTA, and 0.1 M NaCl). The cross-linking reaction was stopped by adding glycine at a final concentration of 0.125 M. Cell lysis and chromatin extraction were performed as previously reported [31]. Chromatin immunoselection was conducted as already described [32, 33] using anti-H3K4me3 (#39159, Lot. 2219006; Active Motif, Carlsbad, CA, USA), anti-H3K9me3 (#39766, Lot. 53843; Active Motif, Carlsbad, CA, USA) and anti-H3K27ac (ab4729, Lot. GR3231887-1; Abcam, Cambridge, UK) antibodies. Immunoselected DNA, once purified and quantified, was preliminary used to test the immunoprecipitation specificity by Real-time qPCR and then processed for libraries preparation and, finally, sequenced in 51 bp single-read mode on a NovaSeq 6000 sequencer (Illumina Inc., San Diego, CA, USA).

Reads pre-processing and alignment was performed as previously reported [34]. Resulting uniquely mapped and properly paired reads of each histone PTM were quantitatively comparable between samples (Supplementary Fig. S1) and were used for all downstream analyses. For each replicate of each histone PTM investigated, a Trimmed Mean of M-values (TMM) normalized count was retrieved into consecutive, non-overlapping 10-kb bins (*n* = 309,579) using DiffBind R package and used to perform principal component analysis (PCA).

Enriched regions (peaks) of H3K4me3 were identify using MACS2 software while H3K9me3 and H3K27ac signals were analyzed with epic2 tools. All identified regions were then used to perform a differential binding analysis by Diffbind R package (v3.2.6) [35].

Stringency in the analysis was obtained by creating a consensus dataset for each condition, including peaks that were shared by at least two replicates of the considered group. Regions were considered differentially enriched (DERs) if the following criteria were met: log2(fold change—FC) ≥ 0.58 and false discovery rate (FDR) ≤ 0.01, for upregulated DERs, while log2(FC) ≤ −0.58 and FDR ≤ 0.01, for downregulated DERs. ChIPseeker R package (v1.28.3) was applied to annotate differentially enriched regions (DERs) while pathway analysis was conducted using the Ingenuity Pathway Analysis (IPA) software from QIAGEN (Hilden, Germany). Pathways with an absolute Z-score >2 were considered. BAM files were used also to generate a BPM (bins per million) normalized bigwig file (a file format for display of dense, continuous data in a genome browser track) using deepTools bamCoverage (v3.5.1) (chromosome X was ignored for normalization). In addition, multiBamSummary tool from DeepTools was used to retrieve the HM signal across TSS (±2.5 kb) of 23,068 genes for H3K4me3 and H3K27ac while the H3K9me3 signal was calculated using regions from 10 kb upstream the TSS up to the TSS of 23,068 genes. Signals were then normalized using edgeR (TMM normalization) and used for GSEA. Where not specified, all plots have been produced using ggplot2 R package.

## Results

### Cell-free chromatin complexes formation and H3K9me3 reorganization mediated by maltonis

In a previous study, maltonis was shown to interfere with the electrophoretic mobility of genomic DNA isolated from previously treated cells, while the electrophoretic migration was re-established after a pre-incubation of DNA with proteinase K [20]. We investigated the hypothesis that maltonis generates cross-linking between DNA and histone proteins using isolated native chromatin [21] exposed to maltonis at the final concentrations of 2 mM and 4 mM. The observed maltonis-dependent increase of DNA in chromatin complexes-containing fraction (Fig. 1B—left), due to the maltonis-induced crosslinks, seems to require an already structured and stable chromatin conformation since the phenomenon was not detectable when free DNA and purified histones were treated together at the same conditions (Fig. 1B—right). However, the extent of maltonis-induced molecular cross-linking was not comparable with that induced by formaldehyde, used as experimental positive control at a 4 mM concentration.

The already reported γ-H2AX incorporation upon maltonis cellular treatment [19] is coherent with the cellular response aimed at removing the molecular adducts and suggests a possible heterochromatin remodulation and H3K9me3 redistribution as already reported in literature [36–38]. Thus, immunoblot analysis was conducted using total lysates of NB4 cells, treated with different concentrations of maltonis, showing a marked reduction of H3K9me3, a repressive histone mark considered crucial in the leukemogenic processes of AMLs (Fig. 1C).

### Modulation of the gene expression profile of NB4 cells upon exposure to maltonis

The high sensitivity of different hematopoietic cellular models to maltonis exposure has been monitored exploiting the NCI-60 assay of the Developmental Therapeutics Program (DTP-NCI/NIH). The NB4 turned out to be one of the most sensitive cell lines when tested using the same experimental condition of the NCI-60 cellular assay (48 h of single dose treatments—Supplementary Fig. S2). Furthermore, both cell survival and cell cycle perturbations of NB4 cells were monitored in a dose-response experiment after 24 h of treatment to define the optimal sublethal conditions for conducting global gene expression studies (Supplementary Fig. S3A, B), that was estimated equal to 10 µM of maltonis.

**Fig. 2:**
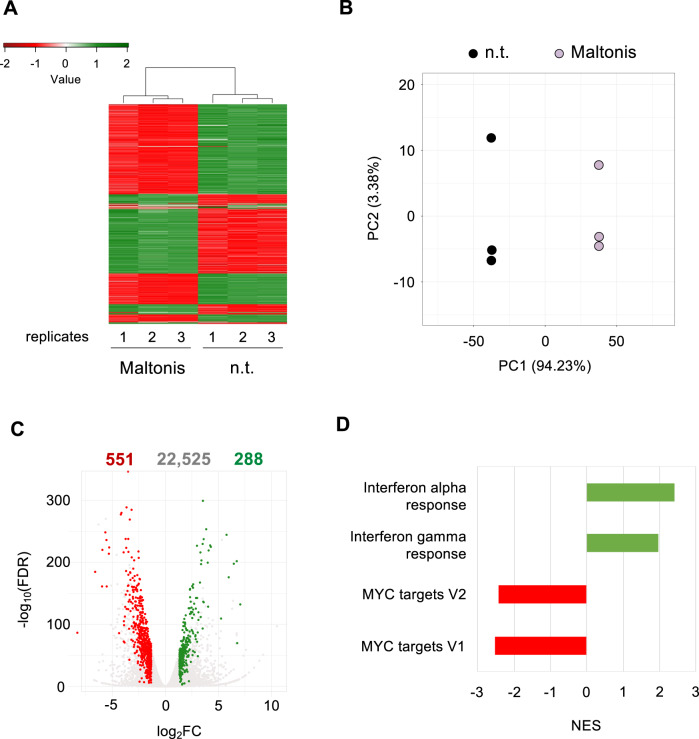
Genome-wide analysis of the gene expression modulations induced by maltonis in NB4 cells. NB4 cells were subjected to maltonis treatment at the concentration of 10 µM for 24 h or left untreated and analyzed by RNA-seq. Biological triplicates were produced for both maltonis-treated and untreated cells. **A** Hierarchical clustering heatmap of the most variable 10% genes between maltonis treated and not treated (n.t.) cells. As reported in the color key, positive Z-scores (green) are associated with higher expression values while negative Z-scores (red) indicate a lower expression value. n.t.: not treated cells. **B** Principal component analysis (PCA) of the most variable 10% expressed genes between not treated (n.t.) and maltonis-treated cells. **C** Volcano plot of differential expressed genes (DEGs) in consequence of maltonis treatments in NB4 cells. Genes from differential gene expression analysis were identified as differentially expressed (DEGs) when the following criteria were met: log_2_(FC) > 1.32, FDR < 0.01, and RPKM_Treat > 10 for upregulated DEGs, while for downregulated DEGs, log_2_(FC) < −1.32, FDR < 0.01, and RPKM_Crtl > 10. Upregulated genes are shown in green while downregulated genes are shown in red. The total number of transcripts analyzed and the one found to be regulated are reported at the top of the graph. **D** Gene set enrichment analysis (GSEA) showing the most upregulated (in green) and downregulated (in red) hallmark gene sets.

RNA-seq was conducted using three biological replicates for both not treated (n.t.) and 10 µM maltonis-treated NB4 cells. After removal of the lowest expressed genes (25%), the overall similarity between samples has been assessed through either a hierarchical clustering (Euclidian distance) and principal component analysis (PCA) using the most variable 10% genes. As reported in Fig. 2, both approaches allowed to cluster the triplicates into distinct groups in function of treatment, suggesting that maltonis had a high impact on gene expression. PCA shows that principal component 1 (PC1), which explains 94.23% of total variability of our dataset, is driven by the treatment, while PC2 (3.38%) variation seems to be related to intragroup variability.

Differential enrichment analysis by edgeR package allowed us to identify genes affected by treatment. We found 551 downregulated and 288 upregulated genes as the most significantly affected by 24 h of 10 µM maltonis treatment, as reported in Fig. 2C (the complete list of regulated genes is reported in Supplementary Table S2).

To understand which pathways or gene networks are affected by maltonis, a gene set enrichment analysis (GSEA) was conducted. As reported in Fig. 2D, GSEA analysis identified as activated gene sets related to IFN-α and IFN-γ response (normalization enrichment score—NES—+2.40 and +1.95, respectively), while gene sets containing c-MYC target genes (hallmark MYC target V1 and hallmark MYC target V2, with NES value of −2.53, −2.41, respectively) were found to be repressed. Similar results were obtained using Ingenuity Pathway Analysis (IPA - data not shown).

### Epigenomic profiling of histone PTM changes induced by maltonis

The global decrease of the H3K9me3 repressive mark observed in maltonis-treated NB4 cells provided the rationale to evaluate the existence of global perturbations of the histone code.

**Fig. 3:**
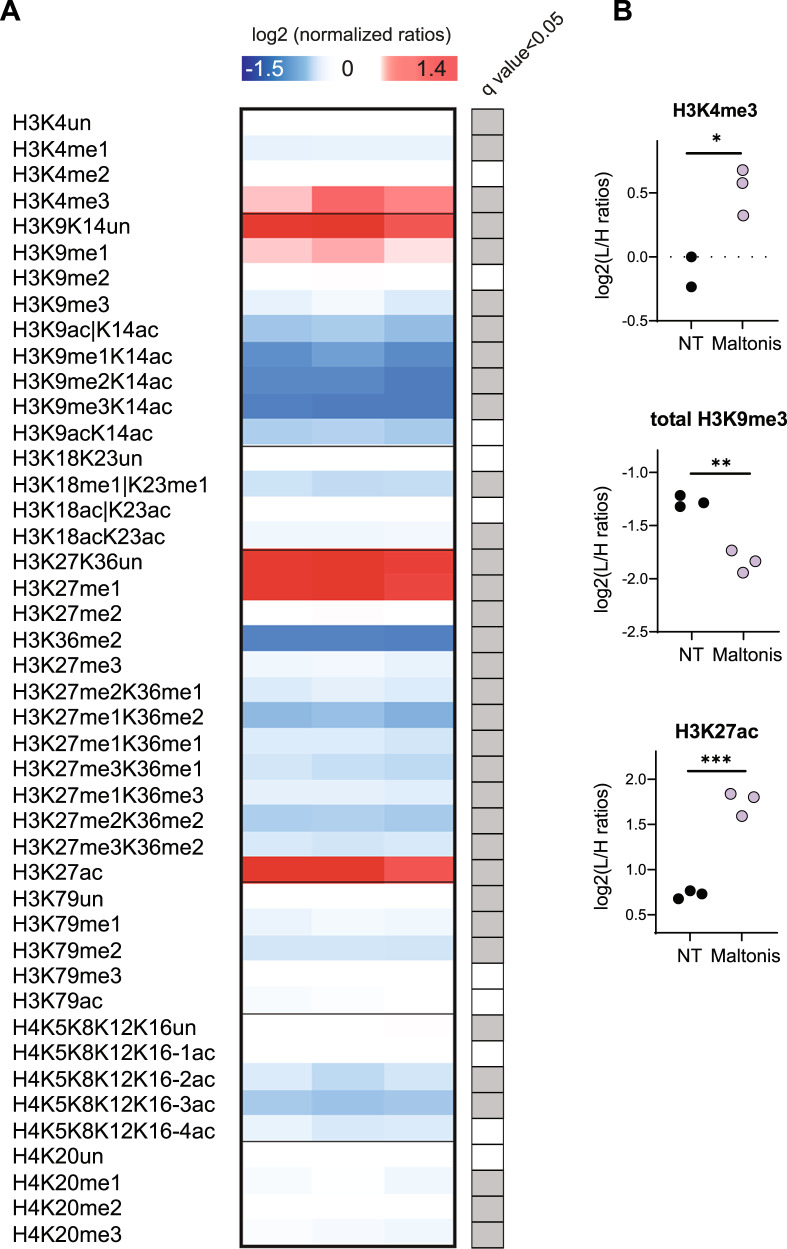
Histone PTMs profiling by mass spectrometry of maltonis-treated NB4 cells. **A** Heatmap display of histone PTMs levels in NB4 cells treated with 10 µM maltonis for 24 h. L/H (light/heavy) relative abundance ratios for differentially modified histone H3 and H4 peptides were obtained using a spike-in strategy (light channel: sample, heavy channel: spike-in standard), and were normalized on the average of the detected signal in the untreated control samples. Significant changes by multiple t-test (*q* value < 0.05) are indicated on the right. L/H ratios for untreated and treated samples are reported in Supplementary Table S1. **B** Interleaved scatter plot display of L/H ratios for untreated and maltonis-treated samples for selected PTMs. Total H3K9me3 indicates the sum of all the peptides containing K9me3 (H3K9me3K14unmod and H3K9me3K14ac). H3K4me3 could be quantified on two out of three replicates in the untreated samples.

**Fig. 4:**
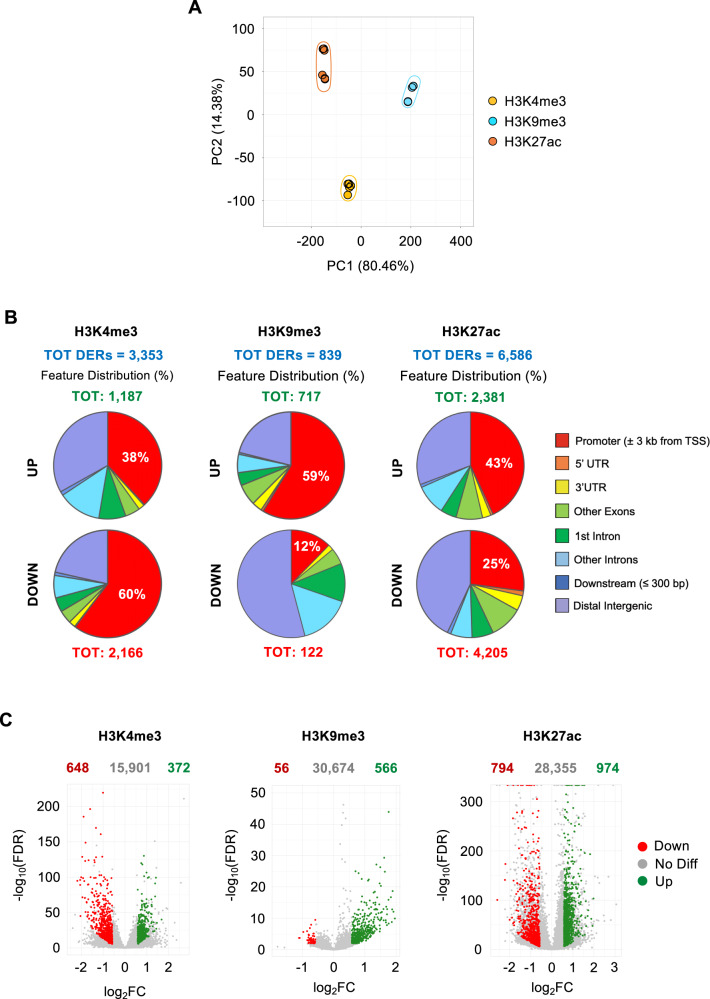
Epigenomic profiling of histone PTMs changes induced by maltonis in NB4 cells. H3K4me3, H3K9me3, and H3K27ac were investigated genome-wide by ChIP-seq in NB4 cells treated with 10 µM maltonis for 24 h or left untreated. Biological triplicates of each treated and untreated sample have been produced and analyzed. **A** PCA showing the clustering of the three fo histone PTMs investigated. **B** Pie charts showing the genomic annotation of DERs found to be enriched (UP) or depleted (DOWN) by maltonis treatment in each histone PTM investigated. The total number of differential enriched regions (DERs) is reported in blue, while upregulated and downregulated regions are indicated in green and red, respectively. **C** Volcano plot showing selected gene-associated DERs. Regions were considered differentially enriched if log2(FC) ≤ −0.58 or log2(FC) ≥ 0.58 and with an FDR ≤ 0.01. Upregulated DERs are shown in green while downregulated DERs are shown in red. The total number of DERs analyzed and the one found regulated are reported at the top of each graph.

We profiled histone PTMs by mass spectrometry (MS) in NB4 cells exposed for 24 h to 10 µM of maltonis (and untreated NB4 cells as control), detecting marked changes in several histone PTMs, in addition to the H3K9me3 decrease (Fig. 3A). These include an increase of H3K4me3, H3K27me1, and H3K27ac and a decrease of H3K14ac, H3K36me2, H3K79 methylation and of acetylations on the histone H4 N-terminal tail. In view of their well-characterized function, together with the observed relevant modulation induced by the treatment (Fig. 3B), we further investigated the genomic distribution of the transcriptionally permissive H3K4me3 and H3K27ac marks, together with the repressive one, H3K9me3. To this aim, ChIP-seq experiments were carried out using chromatin isolated from the same samples used for RNA-seq and mass spectrometry analyses (untreated and 10 µM maltonis-treated NB4 cells) and H3K9me3, H3K4me3, and H3K27ac as bait for immunoprecipitation. PC1 and PC2 plotted in Fig. 4A show that the samples cluster in function of the epigenetic mark investigated. In addition, PCA relative to each histone PTM clearly identifies clusters correlated to maltonis treatment (Supplementary Fig. S4A, B), confirming the ability of maltonis to affect the landscape of all histone PTMs investigated.

A differential enrichment analysis was performed for each histone PTM, identifying depleted and enriched regions. In particular, DiffBind analysis identified 3353 differentially enriched regions (DERs) for H3K4me3, 839 DERs for H3K9me3, and 6586 DERs for H3K27ac. DERs annotation was then performed using ChIPseeker (Supplementary Fig. S5) and results, showing enriched and depleted DERs, are reported in Fig. 4B. Regarding H3K4me3, we found that 38% (*n* = 452) and 60% (*n* = 1306) of DERs of enriched and depleted DERs, respectively, are found in promoter regions (−3 kb to +3 kb from the TSS). Interestingly, H3K27ac showed an opposite behavior, with 43% (*n* = 1026) annotated as promoters in upregulated DERs and only 25% (*n* = 1085) in depleted DERs. Moreover, the repressive mark H3K9me3 showed the greatest difference between depleted and enriched promoters, with an increase observed in 421 promoters (59% of enriched DERs), and a depletion in only 15 promoters (12% of depleted DERs). Genes showing the higher and most significant enrichment or depletion are reported in the volcano plots in Fig. 4C. The lists of genes showing histone PTM regulation are reported in Supplementary Table S3, Table S4, and Table S5 for H3K4me3, H3K9me3, and H3K27ac, respectively.

**Fig. 5:**
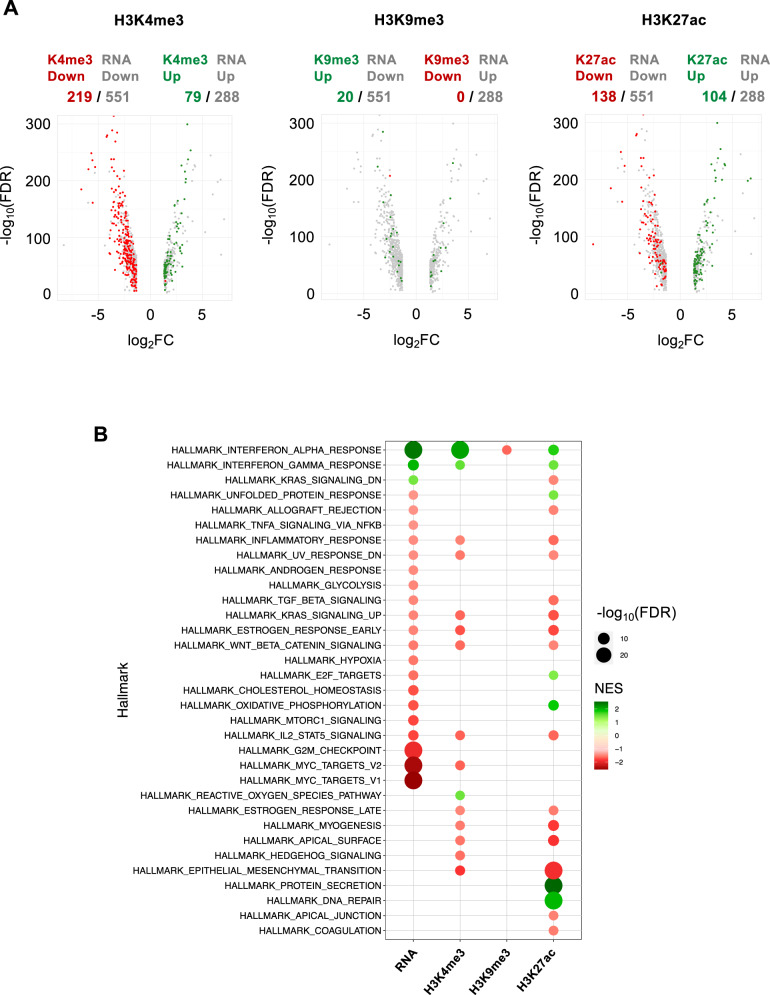
Genome-wide comparison between gene expression and epigenomic modulations in NB4 cells treated with maltonis. **A** Volcano plots showing the distribution of epigenetically modulated genes as compared with their differential expression status (DEGs) for each histone PTM investigated. DEGs that are not epigenetically modified are indicated in gray, while DEGs showing increased or decreased histone PTMs modulation are shown in green and red, respectively. The number of epigenetically upregulated and downregulated genes as compared with upregulated and downregulated DEGs is reported at the top of each graph. **B** GSEA of DEGs and genes with histone PTMs modulations. Hallmarks showing increased normalized enrichment score (NES) are indicated in green while the one with a negative NES are shown in red. Dimension of dots reflect FDR values as indicated in figure.

Then, we compared gene expression data from RNA-seq with the observed epigenomic modulations, finding a strong association between changes of H3K4me3 and H3K27ac signal at gene promoters and related transcripts levels: most of the promoters found to be enriched by these histone PTMs belong to genes showing an increased transcription rate (green dots), while genes whose promoter is characterized by depletion of these histone PTMs due to maltonis exposure showed a decreased expression (red dots—Fig. 5A). Unexpectedly, no correlation was found between the distribution of the H3K9me3 repressive histone modification and DEGs (Fig. 5A—central volcano plot).

The biological significance of the observed histone PTMs modulations was also investigated by GSEA, showing a concordance between H3K4me3, H3K27ac and gene expression changes (Fig. 5B). In particular, H3K4me3 confirmed the most significant pathways already identified using the differentially expressed genes (DEGs), namely the upregulation of alpha and gamma interferon response and the downregulation of c-MYC pathway (Fig. 5B). Other downregulated signaling pathways, such as IL2-STAT5, WNT beta catenin, early estrogen response, genes upregulated by KRAS (KRAS-UP) and those involved in inflammatory response, were also identified as concordant with gene expression GSEA analysis. Regarding H3K27ac, alpha and gamma interferon responses were also identified as upregulated pathways, together with others such as DNA repair, unfolded protein response and protein secretion, and E2F targets; even in this case, pathways such as IL2-STAT5, WNT beta catenin, early estrogen response, KRAS-UP, TGF-beta signaling, and epithelial–mesenchymal transition were found downregulated and in accordance with H3K4me3 and/or gene expression data. Although only the interferon alpha response gene set was identified as affected by H3K9me3 changes by GSEA, the behavior of this transcriptionally repressive mark is in line with the changes observed in gene expression and in the other histone PTMs.

### c-MYC is a master regulator of maltonis-induced biological response of NB4 cells

**Fig. 6:**
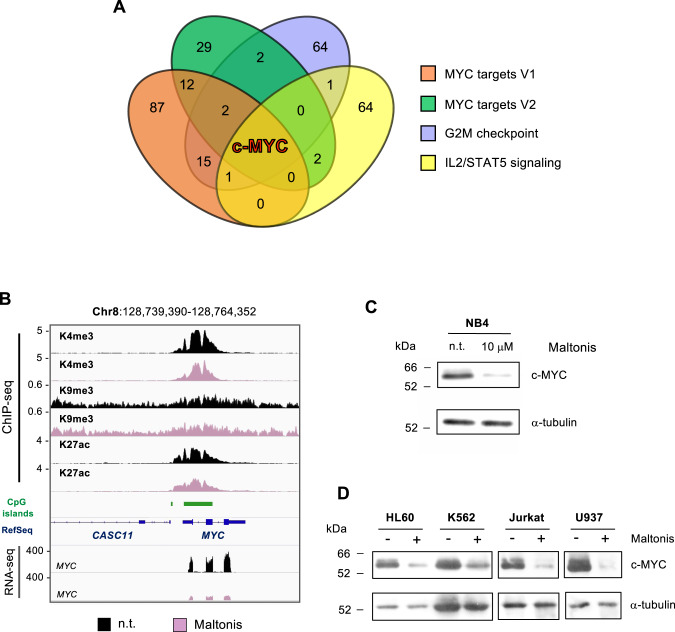
C-MYC as a master regulator of maltonis-induced biological response in NB4 cells. **A** Venn diagram of genes that contribute to the NES of the main downregulated hallmarks gene sets identified by GSEA. **B** Snapshots showing ChIP-seq and RNA-seq signals at *MYC* gene locus. Signals from untreated NB4 cells are indicated in black, while those from maltonis-treated cells are in lilac. RefSeq genes and CpG islands are shown in blue and green, respectively. **C** Evaluation of changes induced by maltonis to c-MYC protein abundance in NB4 cells. Cells were treated with the reported concentrations of maltonis and total cell lysates were analyzed by western blot. **D** Evaluation of changes induced by maltonis to c-MYC protein abundance in different hematopoietic tumor cell lines.

Concomitantly with the theoretically expected antiviral-like response, triggered by the maltonis-induced DNA-histone cross-linking, we found that c-MYC can be considered the master regulator of most of the downregulated pathways identified by GSEA (Fig. 6A).

The maltonis-mediated downregulation of *MYC* expression, observed by RNA-seq (−3.6 folds) is associated with a slight (−1.44 folds) but significant (FDR = 2.98E−35) decrease of H3K4me3 at the *MYC* promoter. Interestingly, neither H3K9me3 nor H3K27ac levels seem to be involved in the regulation of *MYC* expression (Fig. 6B).

The decreased *MYC* expression measured at mRNA level was then confirmed at the protein level in NB4 cells (Fig. 6C). Notably, similar results were obtained in other hematopoietic tumor cell lines, such as HL60, K562, Jurkat, and U937 (Fig. 6D). Comparable decrease of *MYC* expression was observed also upon treatments with cytotoxic doses of maltonis in NB4 cells (50 µM and 100 µM, Supplementary Fig. S6A).

## Discussion

In this study we show that maltonis triggers a massive epigenetic reprogramming of APL cells. The epigenomic reorganization has been extensively characterized following both the transcription permissive H3K4me3 and H3K27ac and the transcription repressive H3K9me3 marks, in function of their important changes measured by quantitative mass spectrometry. H3K9me3 is known to be reorganized as part of the cellular stress response and during γ-H2AX incorporation, and it has been studied here because of the ability of maltonis to target DNA-protein structure and to recruit γ-H2AX. Bulk levels of H3K9me3 decreased upon cell exposure to maltonis. This observation is apparently in contrast with the higher number of genes found downregulated by RNA-seq (*n* = 551), respect to those found upregulated (*n* = 288). In addition, H3K9me3 decrement does not seem to be functionally linked with gene expression, since the distribution of H3K9me3 DERs did not correlate with the modulation of gene transcripts. In fact, among the 566 DERs in which H3K9me3 is increased by maltonis, only few (*n* = 20) involve repressed genes. The apparent paradox of the massive global reduction of H3K9me3 upon maltonis treatment, concomitant with a higher number of genomic regions characterized by an increase of this mark (*n* = 566), with respect to those with a decreased H3K9me3 (*n* = 56), can be explained by the reduction of immunoprecipitated heterochromatin regions. In fact, the DNA obtained by immunoselection of H3K9me3 revealed, once analyzed by Real-Time qPCR, a maltonis-dependent decrement of the amount of alphoid (centromeric) and satellite-2 (pericentromeric) DNA, heterochromatin regions normally characterized by high H3K9me3 levels (Supplementary Fig. S6B, C) [39]. Other authors [6, 40] have associated the genomic redistribution of H3K9me3, and a massive decreased level at heterochromatin repressive regions, with a rapid de-silencing of transposable elements (TEs) that can induce an immune response or, potentially, a genomic instability impacting on cancer cell survival. In accordance with these observations, we found that 91.79% (*n* = 358) of TE families affected by maltonis treatment are upregulated, while only 8.2% (*n* = 32) are downregulated (Supplementary Fig. S7A, B and Table S6). In addition, we found that LTR families, which are the majority (69.55%) of the TE families found upregulated, show a decrease of H3K9me3 signal (Supplementary Fig. S7C). The maltonis-dependent upregulation of IFN-α and -γ antiviral response and the concomitant decreased cell survival and cell cycle modulation (Supplementary Fig. S3A, B) further confirm this view and is in line with recent observation that inhibition of both EZH2 and G9a histone methyltransferase activities suppresses cell proliferation in multiple myeloma, through the induction of an interferon response [41, 42].

As expected, maltonis-mediated changes of H3K4me3 and H3K27ac signals are strongly associated with the global elaboration of gene expression. In fact, most of the genes found upregulated are characterized by higher levels of H3K4me3 and H3K27ac at their promoter (green dots of Fig. 5A); on the contrary downregulated genes are often characterized by decreased levels of both histone marks (red dots of Fig. 5A).

Gene expression analysis identified 551 downregulated genes and 288 upregulated genes and subsequent GSEA analysis identified, in addition to the immune-like response, different downregulated cellular pathways (G2M checkpoint—coherent with G2/M arrest observed—, KRAS-UP signaling, epithelial–mesenchymal transition and others) among which c-MYC targets V1 and c-MYC targets V2 represent the most significant (for the complete list of pathways see Fig. 5B).

Importantly, c-MYC represents the common denominator of the downregulated gene set, as reported in Fig. 6A, and the epigenetic regulation of its expression was further investigated.

Noteworthy, c-MYC is one of the most frequently deregulated driver genes in human cancer and for decades it has been investigated as a possible target of novel therapeutic approaches. c-MYC is required to maintain the balance between self-renewal and differentiation of hematopoietic stem cells (HSCs); not surprisingly, c-MYC is commonly overexpressed in both solid and hematopoietic malignancies (both lymphoid and myeloid neoplasms). In particular, c-MYC was found to be overexpressed in AML, both in the presence and in the absence of a chromosomal translocation, as well as in pediatric subtypes and those forms related to cytotoxic therapy [43–46], and its role in AML development has been proved in vivo [47, 48].

The analysis of the epigenetic status of c-MYC regulatory region revealed a slight but significant decrease of H3K4me3 signal at c-MYC locus while neither H3K27ac nor H3K9me3 levels changed upon treatment (Fig. 6B). Nevertheless, at this stage, the involvement of H3K4me3 in the reduced expression of c-MYC upon maltonis can be only hypothesized. The modulation of c-MYC expression, monitored at the mRNA level, was further investigated by western blot confirming a robust protein downregulation not only in NB4 cells, but also in other hematopoietic neoplasm cellular models (HL-60, K562, Jurkat, and U937—Fig. 6D). Noteworthy is the observation that the most sensitive cell lines (hematopoietic and colon cancers) are those characterized by higher c-MYC expression levels (Supplementary Fig. S8).

Taken together, the data reported here demonstrate the ability of maltonis to epigenetically reprogram NB4 cells (AML-M3) inducing an elaboration of the gene expression able to arrest the cell cycle and suggesting an intriguing antiviral-like response and the downregulation of c-MYC-related pathways. It is possible that the global reduction of the H3K9me3 signal, considered part of the leukemogenic mechanisms in AML, induces a deconstructing effect towards the centromeric (alphoid DNA) and pericentromeric (satellite 2) heterochromatin that could be responsible for an antiviral-like response and increased genomic instability. Regarding this last point, and in function of the already known crosstalk between H3K9me3 and DNA methylation in heterochromatin formation [49, 50], together with the observation of a robust DNMT3b downregulation induced by maltonis, it is not surprising now the observation of an in vitro synergic biological effect between maltonis and DNA demethylating agents, such as 5-aza-cytidine (AZA) and 5-aza-2’-deoxycytidine (DAC), against hematopoietic cancer models [51] that supports the therapeutic potential of maltonis not only against AML neoplasms, but also other types of hematopoietic disorders.

## Supplementary information

Supplemental Figure S1

Supplemental Figure S2

Supplemental Figure S3

Supplemental Figure S4

Supplemental Figure S5

Supplemental Figure S6

Supplemental Figure S7

Supplemental Figure S8

Supplemental Table Legend

Supplemental Table 1

Supplemental Table 2

Supplemental Table 3

Supplemental Table 4

Supplemental Table 5

Supplemental Table 6

## Data Availability

ChIP-seq and RNA-seq data have been deposited with accession number GSE205346 on GEO repository (https://www.ncbi.nlm.nih.gov/geo/).
