# Supplementary material for: Small molecule-induced epigenomic reprogramming of APL blasts leading to antiviral-like response and c-MYC downregulation
Source: Cancer Gene Ther. 2022 Dec 19;30(5):671–82. doi: 10.1038/s41417-022-00576-w (PMC10191840; doi:10.1038/s41417-022-00576-w)
Supplement: Supplementary file 1 — Supplemental Figure S1 [file 41417_2022_576_MOESM1_ESM.pdf]

SUPPL. FIGURE S1

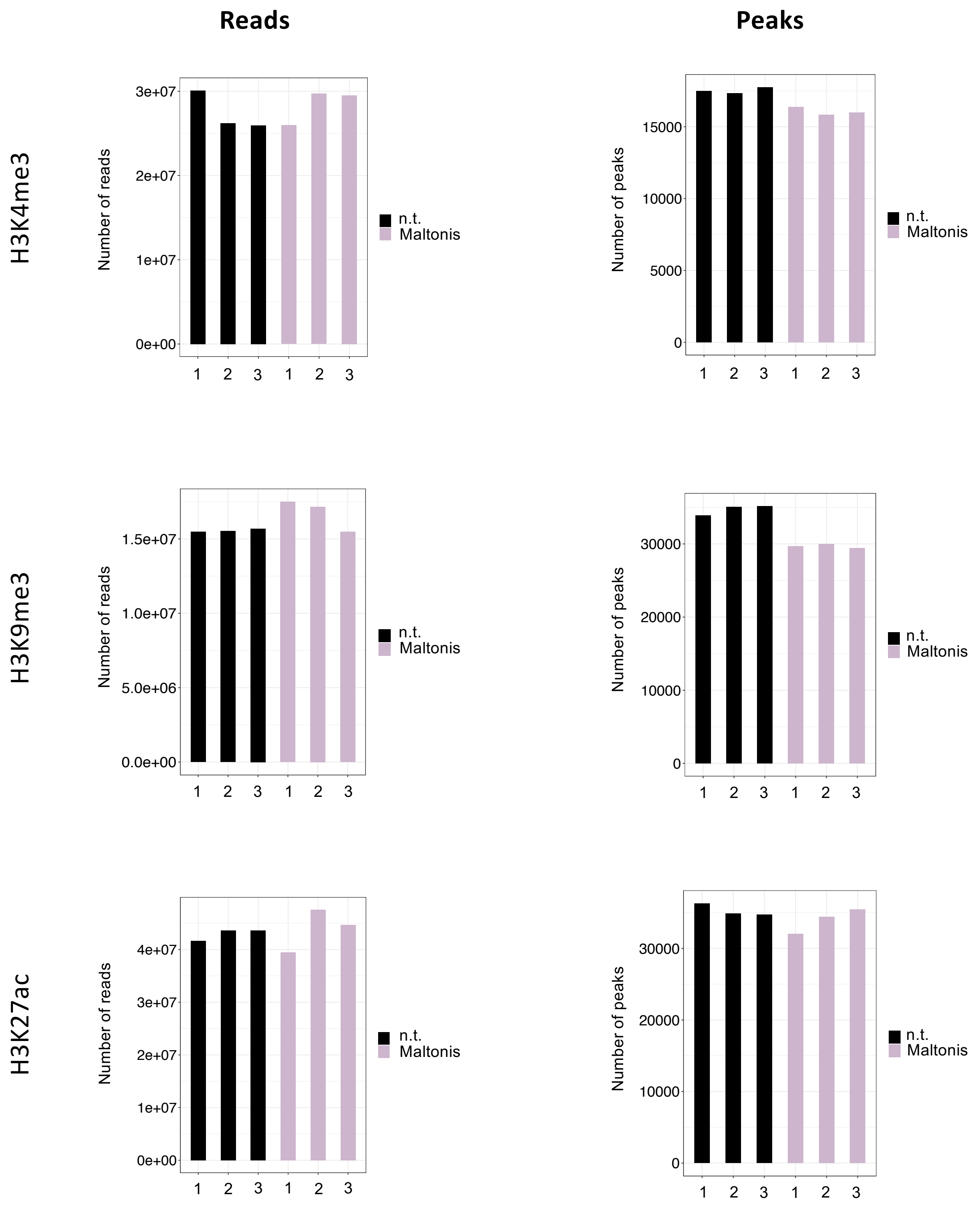


**Supplementary Figure S1. Number of final reads and peaks identified for each histone PTM.** Histograms showing the number of unique and properly paired reads (left) and the number of called peaks (right) for each histone PTM investigated in the study in not treated (n.t. – black bars) and maltonis-treated (lilac bars) NB4 cells (three replicates for each condition). The numbers (1, 2, 3) indicates replicates.
