# Supplementary material for: Small molecule-induced epigenomic reprogramming of APL blasts leading to antiviral-like response and c-MYC downregulation
Source: Cancer Gene Ther. 2022 Dec 19;30(5):671–82. doi: 10.1038/s41417-022-00576-w (PMC10191840; doi:10.1038/s41417-022-00576-w)
Supplement: Supplementary file 2 — Supplemental Figure S2 [file 41417_2022_576_MOESM2_ESM.pdf]

SUPPL. FIGURE S2

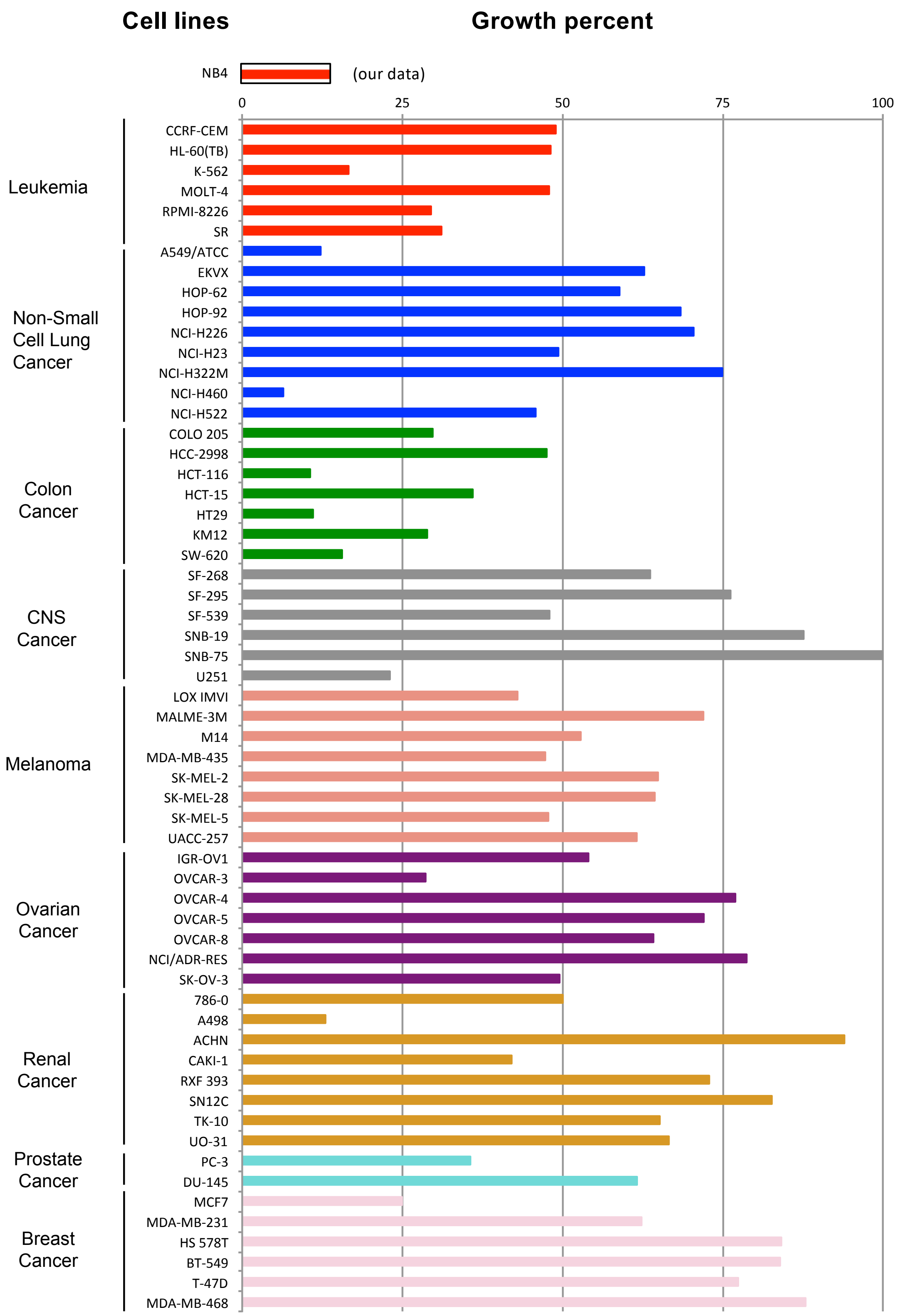

Supplementary Figure S2. Effect of maltonis on the growth of various cancer cell lines. Maltonis was selected and tested in the Developmental Therapeutics Program (DTP) of the National Cancer Institute (NCI/NIH). NCI-60 human tumor cell line anti-cancer drug screening is based on the treatment of 60 different cancer cell lines at the concentration of 10 µM for 48 hours. Data are reported as percentage of cell growth respect to untreated cells. Analyses were done as described at http://dtp.cancer.gov/discovery_development/nci-60. NB4 cells (APL model) were cultured and treated at the same conditions (our data) used in the NCI-60 protocol and added to the graph.
