# Supplementary material for: Small molecule-induced epigenomic reprogramming of APL blasts leading to antiviral-like response and c-MYC downregulation
Source: Cancer Gene Ther. 2022 Dec 19;30(5):671–82. doi: 10.1038/s41417-022-00576-w (PMC10191840; doi:10.1038/s41417-022-00576-w)
Supplement: Supplementary file 3 — Supplemental Figure S3 [file 41417_2022_576_MOESM3_ESM.pdf]

SUPPL. FIGURE S3

A

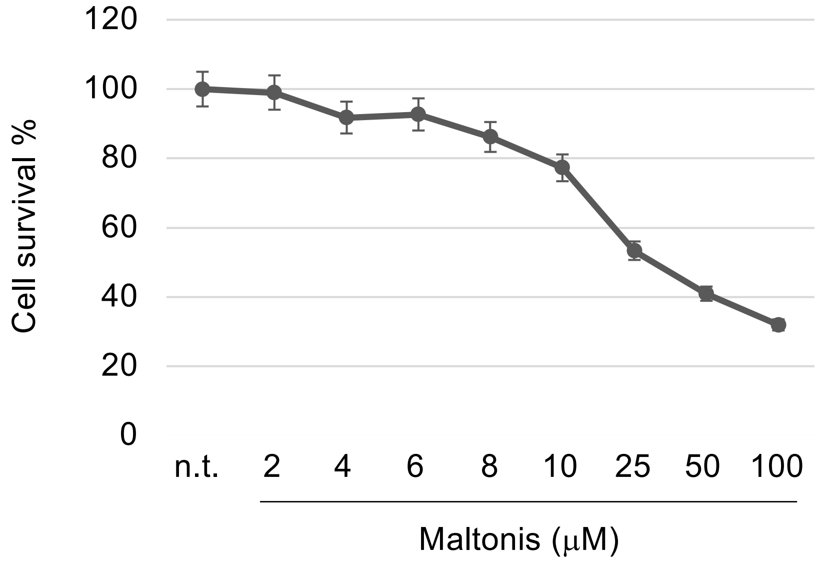

B

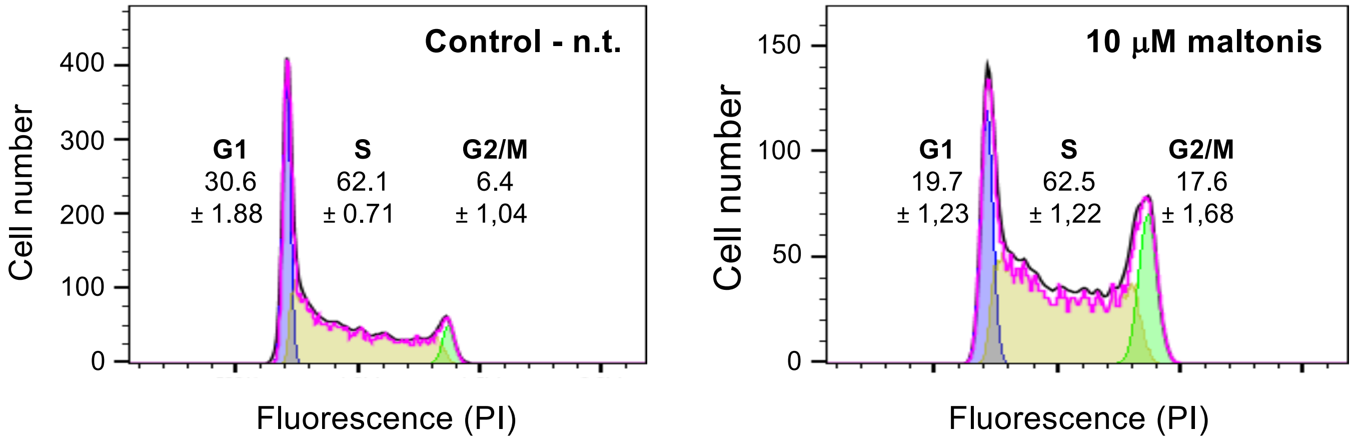

Supplementary Figure S3. Dose-response and evaluation of cell cycle perturbations induced by maltonis in NB4 cells. A. Cell survival (evaluated by trypan blue exclusion assay using the Cell Drop FL automatic cell counter by Denovix – Wilminton, DE, USA) of NB4 cells after 24 hours of maltonis exposure at the indicated final concentrations. **B**. NB4 cell cycle perturbations induced by maltonis treatment at the final sublethal concentration of 10 µM. Cells were fixed with ice cold 70% ethanol and stained with propidium iodide (PI) staining solution (0.1% Triton X-100, 50 µg/mL PI, 250 µg/mL RNAse A in PBS). Samples were acquired using BDAccuri C6 Plus flow cytometer (BD Bioscience, Mountain View, CA, USA). Data were analyzed using FlowJo 10.7 software.
