# Supplementary material for: Small molecule-induced epigenomic reprogramming of APL blasts leading to antiviral-like response and c-MYC downregulation
Source: Cancer Gene Ther. 2022 Dec 19;30(5):671–82. doi: 10.1038/s41417-022-00576-w (PMC10191840; doi:10.1038/s41417-022-00576-w)
Supplement: Supplementary file 4 — Supplemental Figure S4 [file 41417_2022_576_MOESM4_ESM.pdf]

SUPPL. FIGURE S4

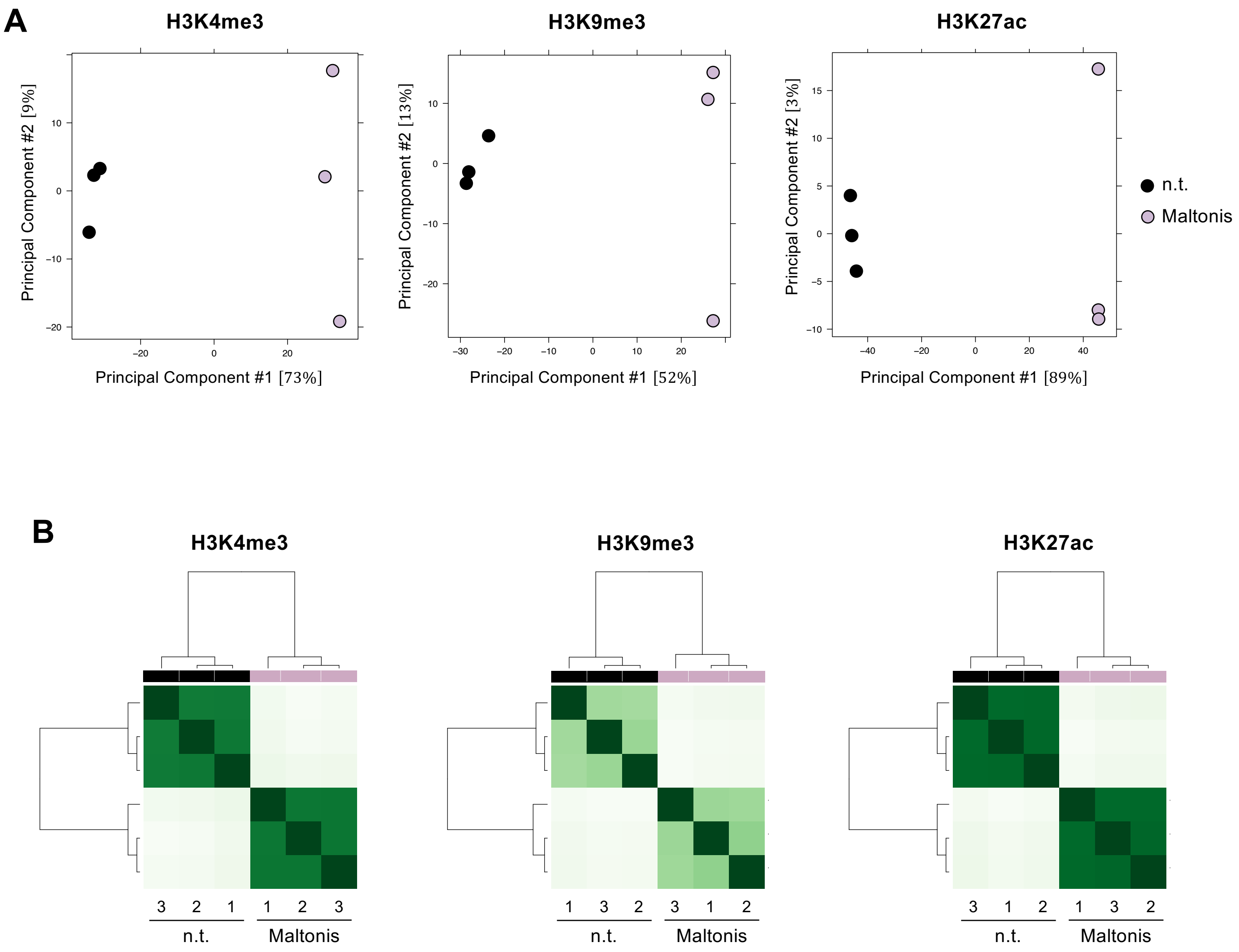

Supplementary Figure S4. Epigenomic profiling of histone PTMs changes induced by maltonis in NB4 cells. H3K4me3, H3K9me3 and H3K27ac were investigated genome-wide by ChIP-seq in NB4 cells treated with 10 µM maltonis for 24 hours or left untreated. Biological triplicates of each treated and untreated sample have been produced and analyzed. **A**. PCA showing the clustering of treated (maltonis) and untreated (n.t.) cells for the three histone PTMs investigated. **B**. Hierarchical clustering heatmap of each histone PTM investigated.
