# Supplementary material for: Small molecule-induced epigenomic reprogramming of APL blasts leading to antiviral-like response and c-MYC downregulation
Source: Cancer Gene Ther. 2022 Dec 19;30(5):671–82. doi: 10.1038/s41417-022-00576-w (PMC10191840; doi:10.1038/s41417-022-00576-w)
Supplement: Supplementary file 5 — Supplemental Figure S5 [file 41417_2022_576_MOESM5_ESM.pdf]

SUPPL. FIGURE S5

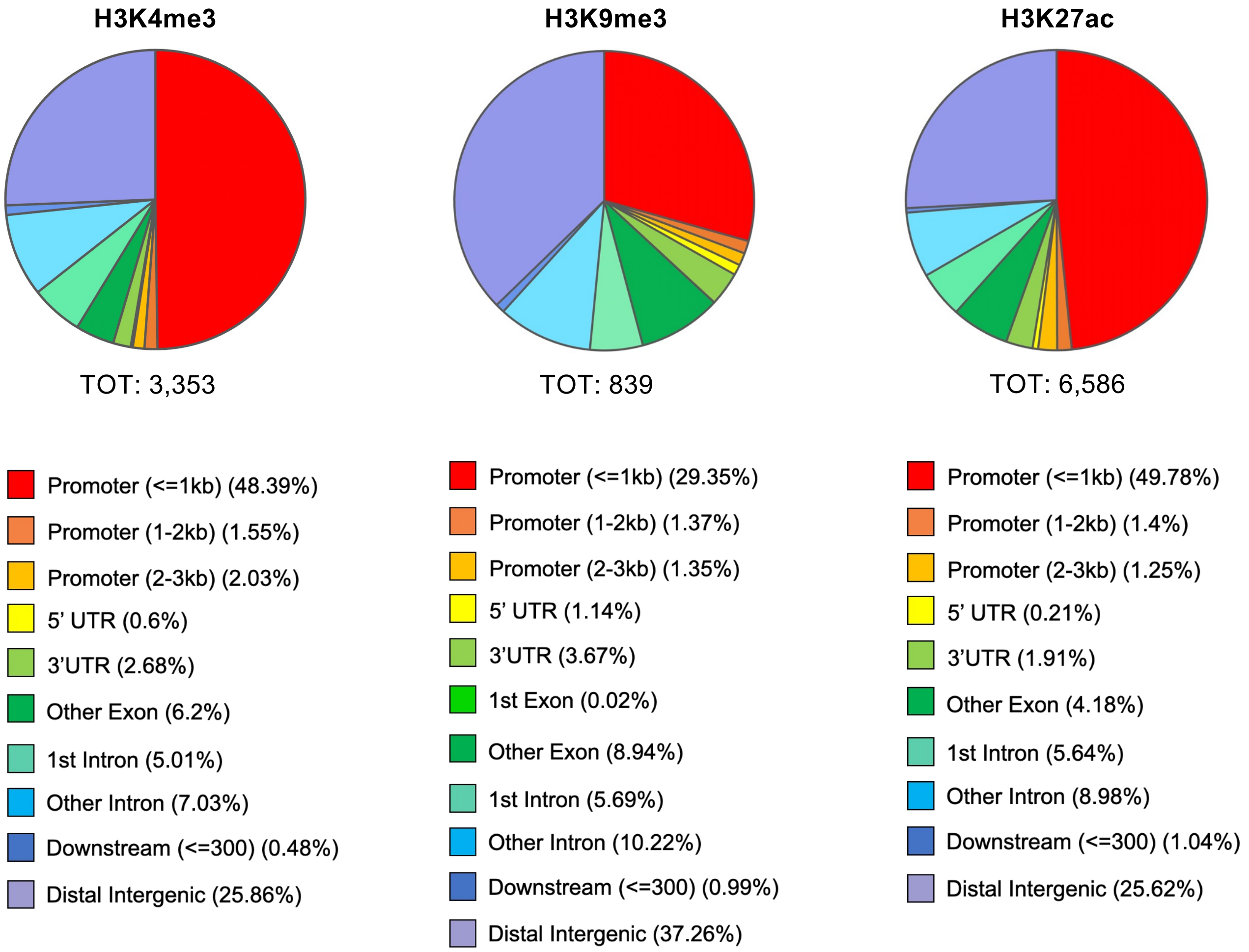

Supplementary Figure S5. Genomic distribution of histone PTMs changes induced by maltonis in NB4 cells. Pie charts showing the distribution across genomic features of the peaks regulated by 24 hours of treatments with 10 µM of maltonis in NB4 cells.
