# Supplementary material for: Small molecule-induced epigenomic reprogramming of APL blasts leading to antiviral-like response and c-MYC downregulation
Source: Cancer Gene Ther. 2022 Dec 19;30(5):671–82. doi: 10.1038/s41417-022-00576-w (PMC10191840; doi:10.1038/s41417-022-00576-w)
Supplement: Supplementary file 6 — Supplemental Figure S6 [file 41417_2022_576_MOESM6_ESM.pdf]

SUPPL. FIGURE S6

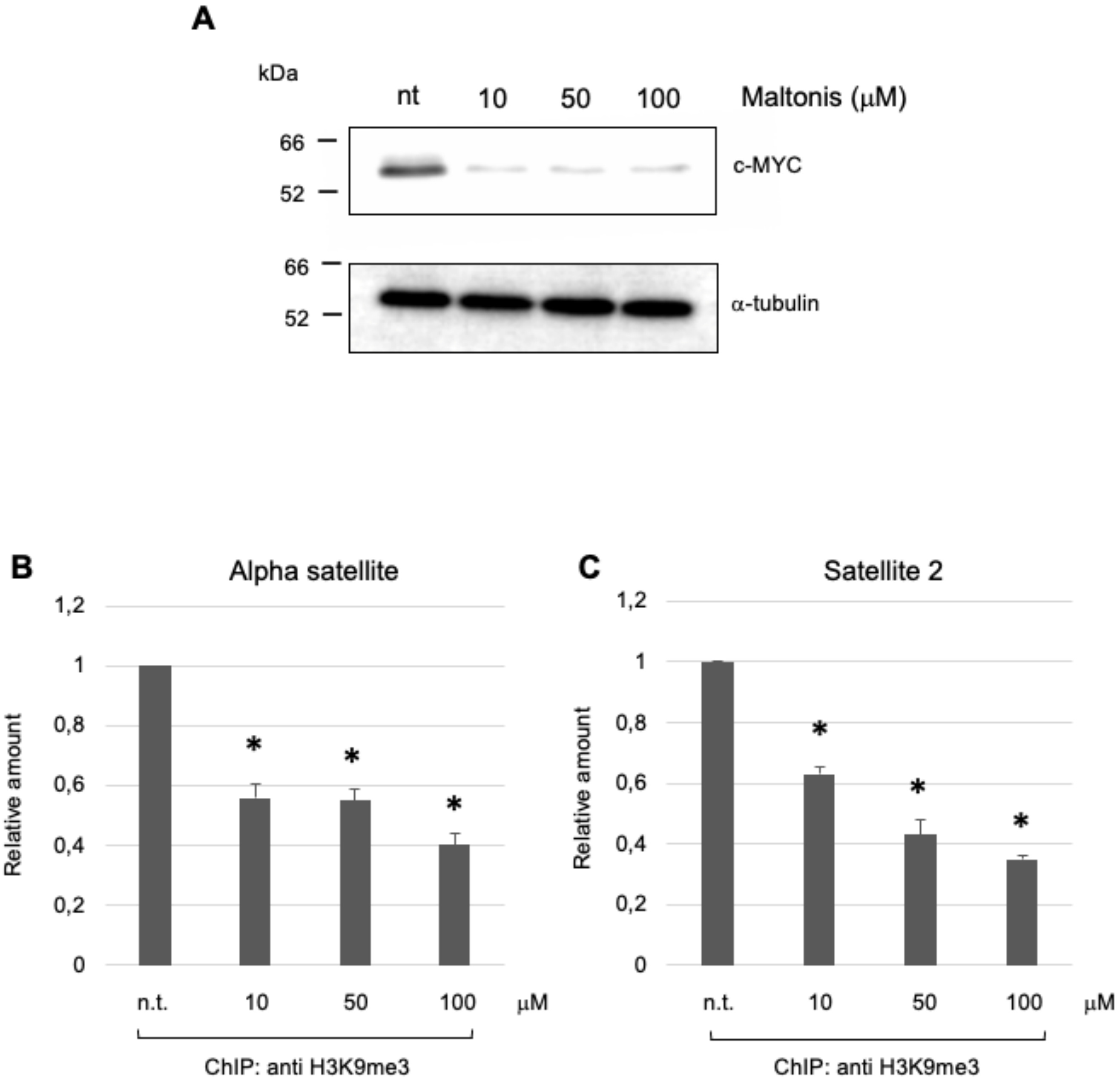

Supplementary Figure S6. Effects of maltonis on c-MYC protein abundance and evaluation of H3K9me3 enrichment at satellite regions. A. Evaluation of changes induced by maltonis on c-MYC protein abundance. NB4 cells were treated with the reported concentrations of maltonis and total cell lysates were analyzed by western blot. **B-C**. The relative enrichment of H3K9me3 at alpha satellite and satellite 2 repetitive sequences was analysed by Real-time qPCR (primer sequences: F-AGCTGAATTCTCAGTAACTTCCTTGTGTTGTGT and R-AGCTGAATCATTCTGACTAGTTTCTATAGG, for alpha satellite; F-ATGGAAATGAAAGGGGTCATCATCT and R-ATTCGAGTCCATTCGATGATTCCAT for satellite 2) on DNA purified from bound fractions of chromatin immunoselected by ChIP using an anti-H3K9me3 antibody (see "Chromatin immunoprecipitation (ChIP)" paragraph of Material and Methods section for full details). * The experiment was conducted in triplicate and the statistical significance of each experimental condition, of the two groups, was evaluated using student's t-test (two-sided), p value < 0.02
