# Supplementary material for: Small molecule-induced epigenomic reprogramming of APL blasts leading to antiviral-like response and c-MYC downregulation
Source: Cancer Gene Ther. 2022 Dec 19;30(5):671–82. doi: 10.1038/s41417-022-00576-w (PMC10191840; doi:10.1038/s41417-022-00576-w)
Supplement: Supplementary file 7 — Supplemental Figure S7 [file 41417_2022_576_MOESM7_ESM.pdf]

SUPPL. FIGURE S7

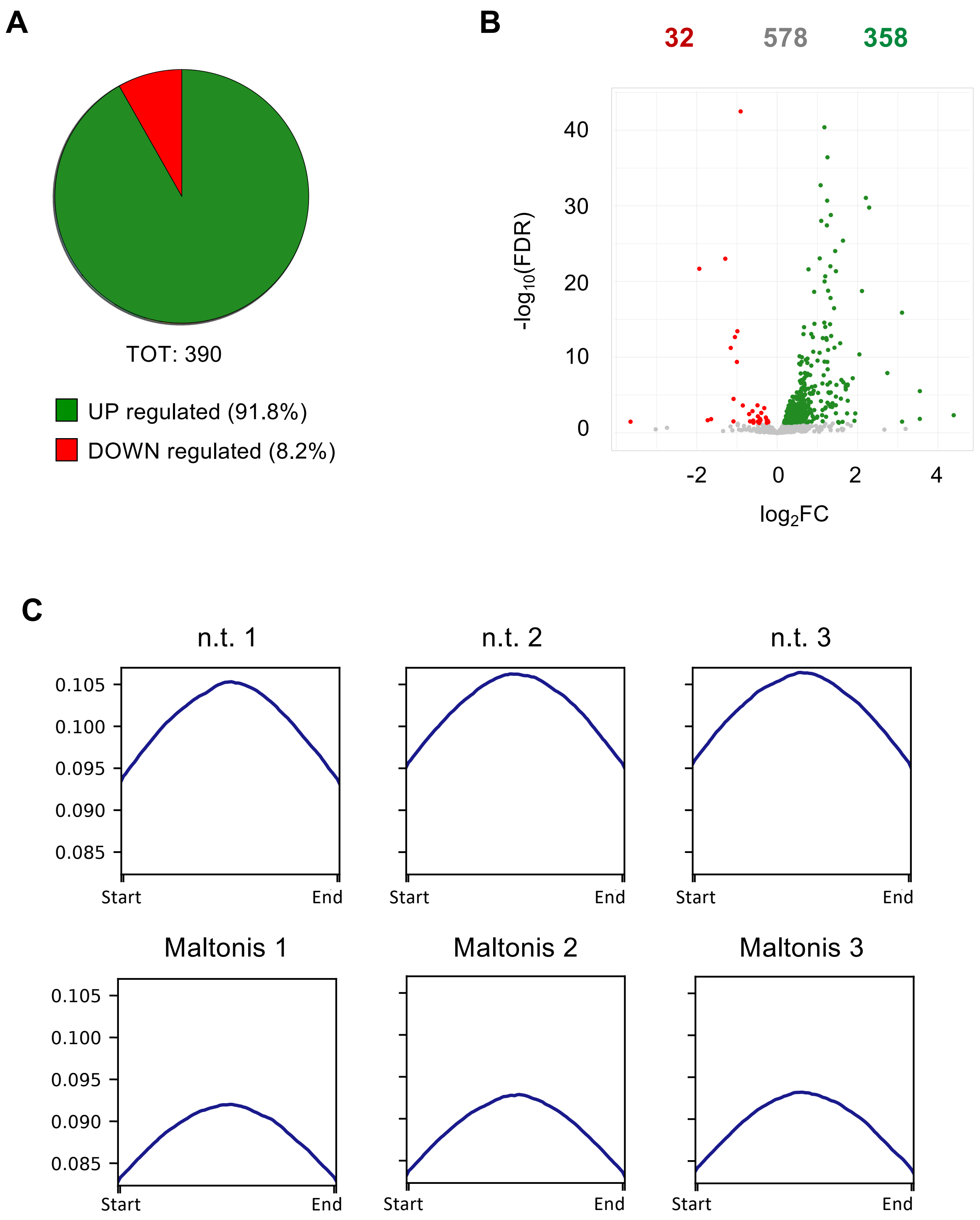

Supplementary Figure S7. Effect of maltonis treatment on transposable elements (TE). RNA-seq data were analysed using TEtranscript, a package including transposable elements in differential expression analysis of RNA-seq datasets (Jin Y. et al., Bioinformatics 2015; 31(22):3593-9) while H3K9me3 signal across long terminal repeat (LTR) was analysed using ComputeMatrix tools from DeepTools. Long terminal repeats annotations were downloaded from https://www.repeatmasker.org/species/hg.html. Missing data were treated as zero. The output of most 20% abundant regions were then plotted using plotHeatmap (DeepTools). **A.** Pie Chart showing the percentage of TE families found to be significantly (FDR ≤ 0.05) upregulated (green) and downregulated (red) by maltonis treatment. **B.** Volcano plot of differential expressed TE in consequence of maltonis treatments of NB4 cells. Upregulated TE (FDR ≤ 0.05) are shown in green while downregulated TE (FDR ≤0.05) are shown in red. TE that are not significantly modulated are indicated in grey. **C.** H3K9me3 mean signal across LTR; The numbers (1, 2, 3) indicates replicates.
