# Supplementary material for: Small molecule-induced epigenomic reprogramming of APL blasts leading to antiviral-like response and c-MYC downregulation
Source: Cancer Gene Ther. 2022 Dec 19;30(5):671–82. doi: 10.1038/s41417-022-00576-w (PMC10191840; doi:10.1038/s41417-022-00576-w)
Supplement: Supplementary file 8 — Supplemental Figure S8 [file 41417_2022_576_MOESM8_ESM.pdf]

SUPPL. FIGURE S8

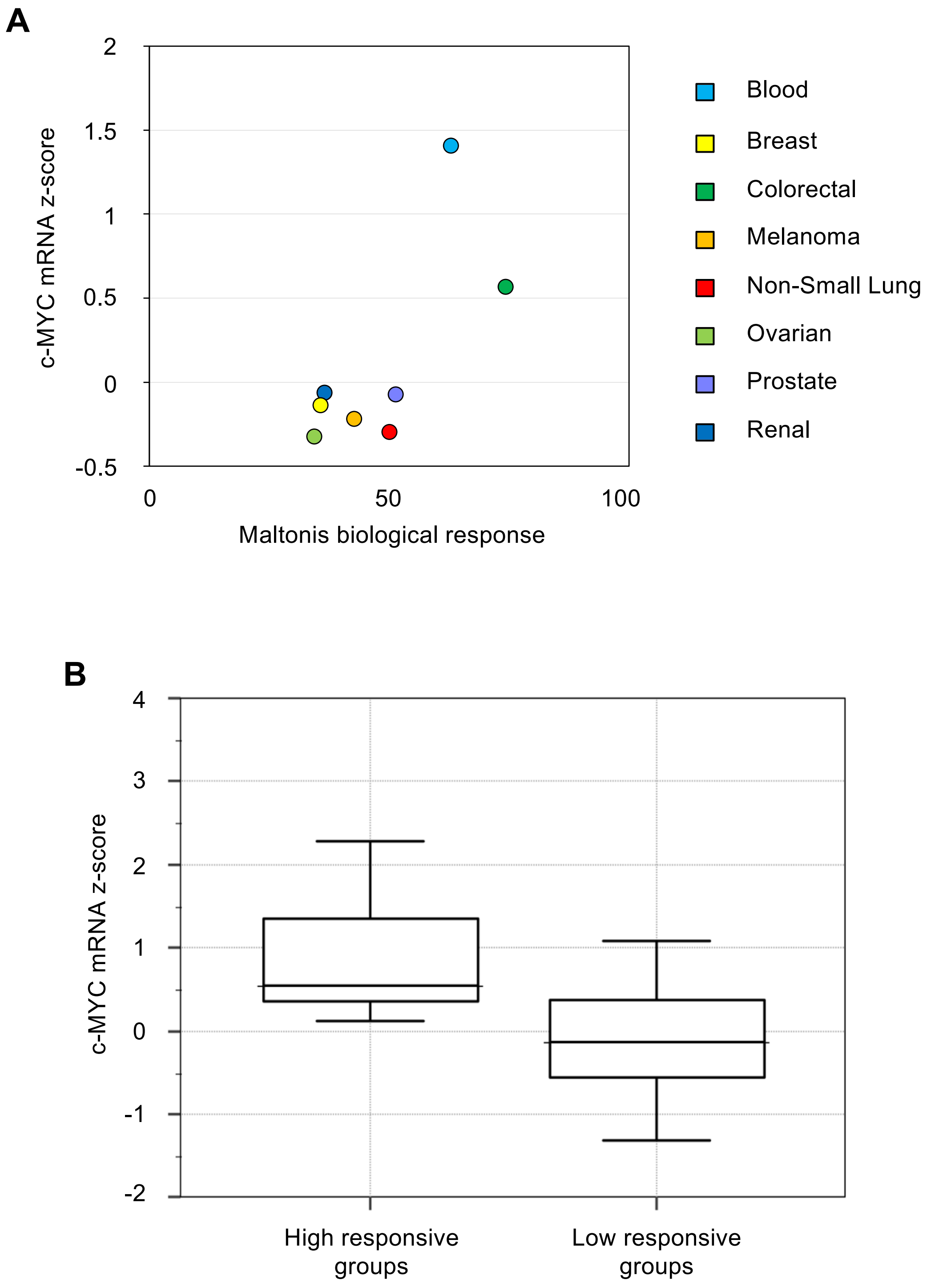

Supplementary Figure S8. Evaluation of c-MYC expression in function of maltonis effect in the NCI60 cancer cell lines. A. Scatter plot of grouped NCI60 cancer cell lines based on mRNA z-score c-MYC expression from the CBioportal (https://www.cbioportal.org) dataset and maltonis effect obtained by the Developmental Therapeutics Program (DTP) of the National Cancer Institute (NCI/NIH). **B**. Boxplot showing grouped cancer cell lines based on mRNA z-score c-MYC expression and maltonis effect (high responsive groups: blood and colorectal cancer cells; low responsive groups: all the other groups). For each box, median are represented with a line across the box. The statistical significance of the difference between the two groups was evaluated using the student's t-test (comparison of means), p value < 0.0001. Statistical analyses were performed using MedCalc for Windows, version 19.4 (MedCalc Software, Ostend, Belgium).
