# Supplementary material for: Small molecule-induced epigenomic reprogramming of APL blasts leading to antiviral-like response and c-MYC downregulation
Source: Cancer Gene Ther. 2022 Dec 19;30(5):671–82. doi: 10.1038/s41417-022-00576-w (PMC10191840; doi:10.1038/s41417-022-00576-w)
Supplement: Supplementary file 9 — Supplemental Table Legend [file 41417_2022_576_MOESM9_ESM.pdf]

Supplemental Tables

Supplemental Tables are available as separate files.

Table S1. Light/Heavy (L/H) ratios of %RA from histone PTMs mass spectrometry analysis.

Table S2. List of genes found regulated by maltonis treatment in NB4 cells by RNA-seq analysis.

FC: fold change; FDR: false discovery rate; RPKM: Reads Per Kilobase of transcript per Million reads mapped; Ctrl: untreated sample; Treat: maltonis-treated sample. Upregulated and downregulated genes are highlighted in green and red, respectively.

Table S3. List of genes showing H3K4me3 modulation upon maltonis treatment in NB4 cells identified by ChIP-seq analysis.

FC: fold change; FDR: false discovery rate; Conc: $\log_2$(Trimmed Mean of M-values); Ctrl: untreated sample; Treat: maltonis-treated sample. Upregulated and downregulated genes are highlighted in green and red, respectively.

Table S4. List of genes showing H3K9me3 modulation upon maltonis treatment in NB4 cells identified by ChIP-seq analysis.

FC: fold change; FDR: false discovery rate; Conc: $\log_2$(Trimmed Mean of M-values); Ctrl: untreated sample; Treat: maltonis-treated sample. Upregulated and downregulated genes are highlighted in green and red, respectively.

Table S5. List of genes showing H3K27ac modulation upon maltonis treatment in NB4 cells identified by ChIP-seq analysis.

FC: fold change; FDR: false discovery rate; Conc: $\log_2$(Trimmed Mean of M-values); Ctrl: untreated sample; Treat: maltonis-treated sample. Upregulated and downregulated genes are highlighted in green and red, respectively.

Table S6. List of transposable elements (TE) found regulated by maltonis treatment in NB4 cells.

Name: Name of the TE family; log2(FC): log2 fold change between the groups; FDR: false discovery rate.
